# Development of Filaments for 3D Printing from Poly(Lactic Acid) Polymeric Nanocomposites and Carbon Nanotubes

**DOI:** 10.3390/polym17172426

**Published:** 2025-09-08

**Authors:** Sanches Ismael de Oliveira, João Carlos Martins da Costa, Nayra Reis do Nascimento, Gilberto Garcia del Pino, José Luis Valin Rivera, Meylí Valin Fernández, José Costa de Macedo Neto

**Affiliations:** 1 Programa de Pós-Graduação em Ciência e Engenharia de Materiais—PPGCEM, Universidade Federal do Amazonas (UFAM), Manaus 69067-005, Amazonas, Brazil; 2 Grupo de Eletroquímica e Materiais Avançados—GEMATA, Departamento de Química, Universidade Federal do Amazonas (UFAM), Manaus 69067-005, Amazonas, Brazil; jcarlosmartins.89@gmail.com; 3 Conecthus Instituto de Tecnologia e Biotecnologia do Amazonas, Avenida Buriti, n°. 3001, Distrito Industrial I, Manaus 69075-000, Amazonas, Brazil; dra.nayrareis@gmail.com; 4 Departamento de Engenharia Mecânica, Universidade do Estado do Amazonas (UEA), Manaus 69050-020, Amazonas, Brazil; gpino@uea.edu.br; 5 Escuela de Ingeniería Mecánica, Pontificia Universidad Católica de Valparaíso, Concepción 2430000, Chile; 6 Department of Mechanical Engineering (DIM), Faculty of Engineering (FI), University of Concepción, Concepción 4070409, Chile; mvalin@udec.cl; 7 Departamento de Engenharia de Materiais, Universidade do Estado do Amazonas (UEA), Manaus 69050-020, Amazonas, Brazil

**Keywords:** nanocomposite, additive manufacturing, DOE, numerical simulation, propellers

## Abstract

The aim of this study is to obtain poly(lactic acid) polymeric nanocomposites and carbon nanotubes for application in drone propellers produced through 3D printing. In this work, a filament based on poly(lactic acid)—PLA/functionalized carbon nanotube (CNT) composites was prepared for the fused deposition modeling (FDM) process. The effects of CNT content **(0.2–1.0%)**, temperature variation, and extruder screw rotation variation were applied in the Design of Experiments (DOE) tool, where the main factors contributing to filament quality, focusing on mechanical strength, were identified. Through this tool, an optimum point for the material’s mechanical strength was reached, showing a value of 48.87 MPa, 43.17% above the initial value of 27.77 MPa. The response surface curve revealed a region where new filaments with similar mechanical strength values to those found in this work could be obtained. The results demonstrate that CNT content, extruder screw rotation, and extruder temperature directly influence filament quality. The data obtained from Thermogravimetry (TG) and Derivative Thermogravimetry (DTG) curves show that the addition of 0.6% CNT by weight does not significantly modify PLA degradation resistance, despite slight differences in temperatures. The main reason for these alterations is the dispersion of CNTs in the PLA matrix and CNT agglomeration. Through the demonstrated simulation, it is possible to confirm the application of the developed material in drone propeller manufacturing, facilitating access and providing new opportunities for users.

## 1. Introduction

The blades of drone propellers are often pieces prone to breakage during imperfect landings or collisions with outdoor objects. The use of modern computational technology, proper design, and material optimization will contribute to constructing a corresponding blade using a 3D printer based on the FDM principle [1].

Fused deposition modeling (FDM) is a 3D printing process where thermoplastic materials are deposited layer by layer through an extrusion nozzle. One of the main advantages of an FDM process is that any geometry of complex shape can be printed directly from a CAD model. One of the most used materials for 3D printing is PLA (polylactic acid) [2].

Recently, filament extrusion and 3D printing technology have been explored for the recycling and upcycling of thermoplastic waste, transforming them into useful composites or nanocomposites. Studies demonstrate the feasibility of using industrial waste from glass-fiber-reinforced polypropylene (GFRPP) and domestic high-density polyethylene (HDPE) waste as the raw material for new filaments, paving the way for a circular economy in additive manufacturing [3].

Due to environmental concerns and the increasing volume of plastic waste in landfills, biodegradable polymers are being extensively studied. One of them is PLA, which has properties comparable to commodity polymers and engineering polymers, but certain characteristics of PLA, such as impact resistance, still need improvement for more specific applications [4].

Biodegradable polymers can be defined as materials that can decompose rapidly, typically within 6 to 24 months, due to the action of microorganisms such as bacteria and fungi, whereas non-biodegradable polymers take hundreds of years to be bioassimilated. Among biodegradable polymers, notable examples include thermoplastic starch, poly(ε-caprolactone), and polylactic acid (PLA) [5,6,7]. It is important to note, however, that under natural conditions, such as in the sea or in landfills without biological control, PLA can take more than 80 years to fully decompose, contributing to plastic and microplastic pollution similarly to conventional plastics.

Nanotechnology is a relatively recent science, discovered by Richard Feynman in 1959, which allows manipulation of atoms on a nanoscale and production of composites with nanoparticles [8,9]. Currently, nanotechnology is one of the forerunners of the Fourth Industrial Revolution. This revolution is a contemporary historical process concerning technological and scientific advancement, characterized by waves of discoveries in various fields of knowledge in a rapid and simultaneous manner [10].

The new applications are associated with various types of nanomaterials, such as nanoparticles, nanocomposites, nanocrystals, carbon-based structures (carbon nanotubes, graphene, quantum dots, and nanofibers), polymeric micelles, etc. [11].

Furthermore, nanomaterials have drawn attention from the scientific community due to properties that can only be achieved when certain materials are on the nanoscale, meaning when dimensions are between 10–100 nm. Examples include graphene, nanotubes, nanodiamonds, platinum, titanium dioxide, gold, silver, copper, hydroxyapatite nanorods, and poly(alkyl-cyanoacrylate) nanoparticles [12].

Within nanomaterials, one of the most prominent materials is carbon nanotubes. Carbon nanotubes (CNTs) are relatively new materials, emerging in the twenty-first century, known for their unparalleled strength compared to others.

CNTs are chosen as reinforcement material in applications like drone propellers due to their unique combination of properties: they are extremely lightweight, possess exceptionally high mechanical strength and stiffness, and have a high aspect ratio (length/diameter), which maximizes load transfer from the polymer matrix. Furthermore, their electrical and thermal properties allow for the development of multifunctional composites, such as integrated strain sensors [13]. The use of recycled fibers, such as carbon fibers, has also gained prominence as a sustainable approach for producing composites [14].

Carbon nanotubes (CNTs) are nanostructures formed by the rolling of one or more sheets of graphene onto themselves, creating cylindrical (tubular) structures with diameters in the nanometer range (nanotubes) and lengths ranging from a few micrometers to several centimeters [15].

When carbon nanotubes are used to form nanocomposites with a polymeric matrix, the resulting product exhibits increased stiffness, toughness, as well as high resistance to thermal shock [16].

## 2. Methodology

**Figure 1:**
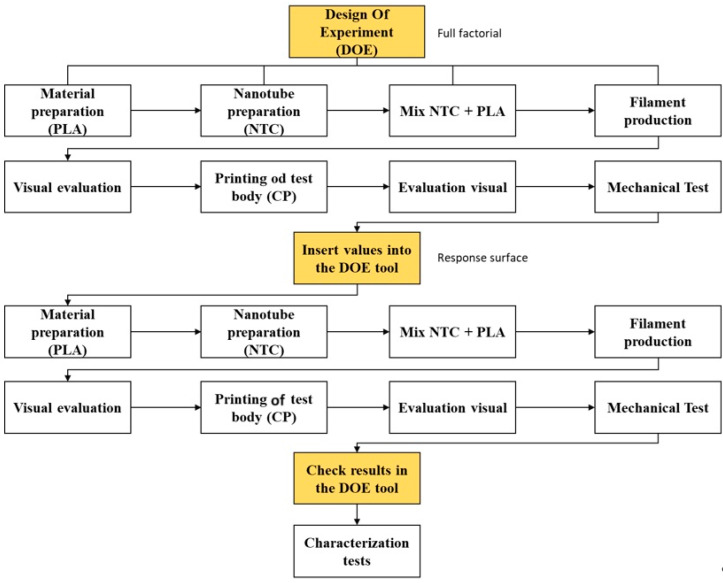
Workflow diagram.

In the flowchart in Figure 1, we can see a step-by-step overview of how the work progressed, demonstrating which steps were completed and in what order.

### 2.1. DOE (Design of Experiments)

The Design of Experiments (DOE) tool was utilized to determine the optimal mixture composition of PLA and CNT for manufacturing the filaments to be used in the test specimens. This statistical tool is commonly employed to identify the best configuration that a set of controllable variables should exhibit to maximize the desired outcome in the process, which is one of the main objectives of this study. Its purpose is to ascertain which independent variables (input variables “X” that influence the quality characteristics of the product/service) have the greatest impact on a given response variable (output and dependent variable “Y”).

In this study, a full factorial experimental design was utilized to determine the most relevant factors that affect the response or not, and response surface methodology was employed to determine the combination of factors to maximize the response. This was done using Minitab^®^ 2023 software, which offers a free 30-day trial version. Such a study is typically conducted when analyzing the influence of all combinations of factors and their respective levels on a response variable of interest, with the aim of optimizing it.

#### 2.1.1. Full Factorial 2^3^

In this research, three factors were determined as input variables, each with two levels, as shown in Table 1. They are:

- CNT content (%CNT): 0.2~1%.
- Filament Extrusion Temperature (ΔT): 190~220 °C (same temperature in all 3 zones).
- Extruder Screw RPM (RPM): 30~60 rpm.

**Figure 2:**
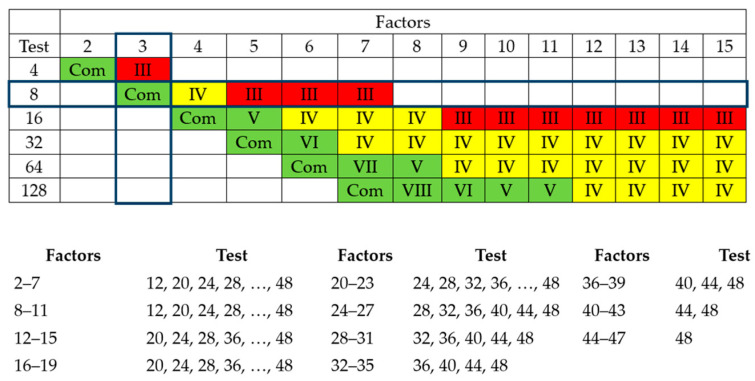
Number of experimental rounds required to achieve a satisfactory result.

With the factors defined, the number of experimental rounds necessary to achieve a satisfactory result was determined. In the image below, we can see that a minimum of 8 trials is required to obtain good results, as shown in Figure 2.

- Green: good results.
- Yellow: reasonable results.
- Red: undesirable results.

The chart illustrates the resolution of the experimental design. For 3 factors, 8 runs (2^3^) result in a full factorial design (indicated by “Com”), which has the highest resolution (V), allowing the analysis of all main effects and interactions without confounding. Lower resolutions (e.g., III, IV) are used for screening factors with fewer runs but confound some effects.

**Figure 3:**
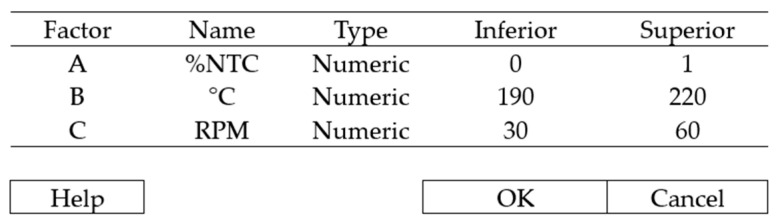
Creating a factorial experiment.

Once the variables were defined, they were input into the Minitab software, as shown in Figure 3.

After completing all the necessary steps, a worksheet was generated, containing the randomized experimentation schedule, as shown in Table 2.

In summary, the generated experimentation schedule indicates that to start the work with a full factorial design, it is necessary to manufacture 11 filaments with the configurations listed in the schedule, varying the CNT content, extruder temperature, and extruder screw rotation.

#### 2.1.2. Response Surface (Box–Behnken)

To initiate the experiment, the same data from the full factorial 23 were utilized:

- CNT content (%CNT): 0.2~1%.
- Filament extrusion temperature (ΔT): 190~220 °C.
- Extruder screw RPM (RPM): 30~60 rpm.

**Figure 4:**
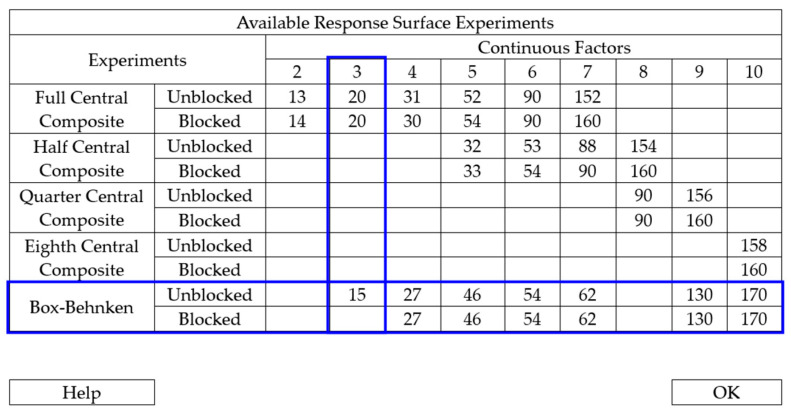
Number of experimental rounds required to obtain a satisfactory result.

Similar to the previous process, the first step involves defining the minimum number of trials to achieve a satisfactory result. As depicted in Figure 4, a minimum of 15 trials are required to conduct the experiment, 7 more than the previous setup.

After completing all necessary steps, a worksheet was generated, Table 3, containing the randomized experimental plan.

In summary, the experimental plan indicates that to begin the work for the response surface, it is necessary to manufacture 15 filaments with the settings outlined in the plan, including variations in CNT content, extruder temperature, and extruder screw rotation.

The values determined for the levels of each factor were based on the literature research, which suggests that we can achieve a considerable improvement in mechanical properties by varying the CNT percentage up to 1%; however, exceeding this value may result in losses.

### 2.2. Manufacturing of Filaments

The nanocomposites were prepared using PLA pellets (PLA Pink L.2CC3N1, 1.75 mm in diameter) from the filament provided by 3DLAB and functionalized CNTs with a diameter of 20 nm and a purity of 90%.

**Figure 5:**
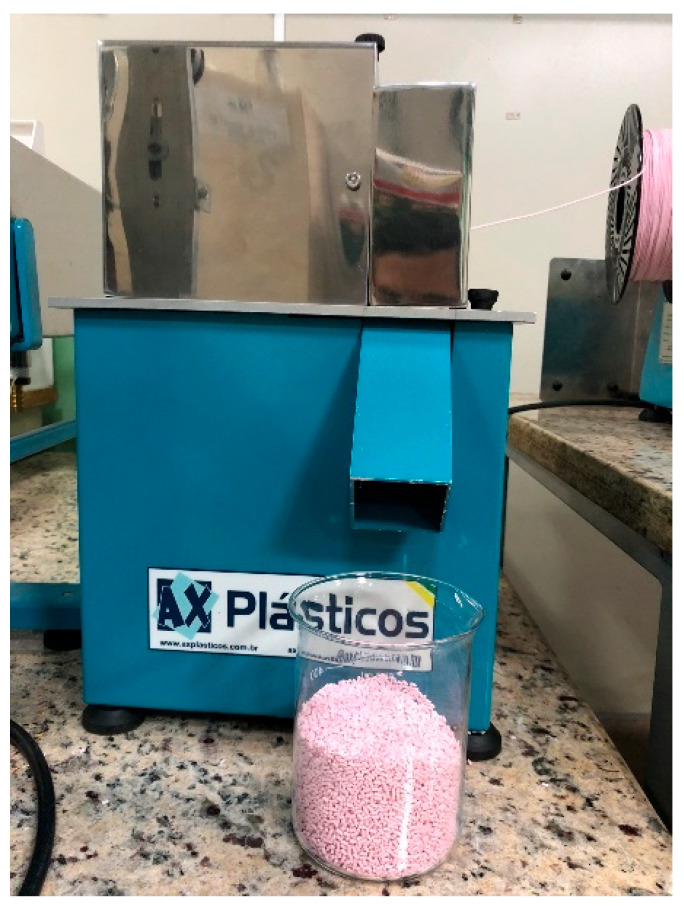
GRAN. 100 AX granulator.

The first step in manufacturing the filaments involved granulating the existing PLA filament. For this purpose, a granulator (GRAN. 100, AX-PLÁSTICO, São Paulo, Brazil) was used, as shown in Figure 5, resulting in pellets with a diameter of 1.0 mm.

**Figure 6:**
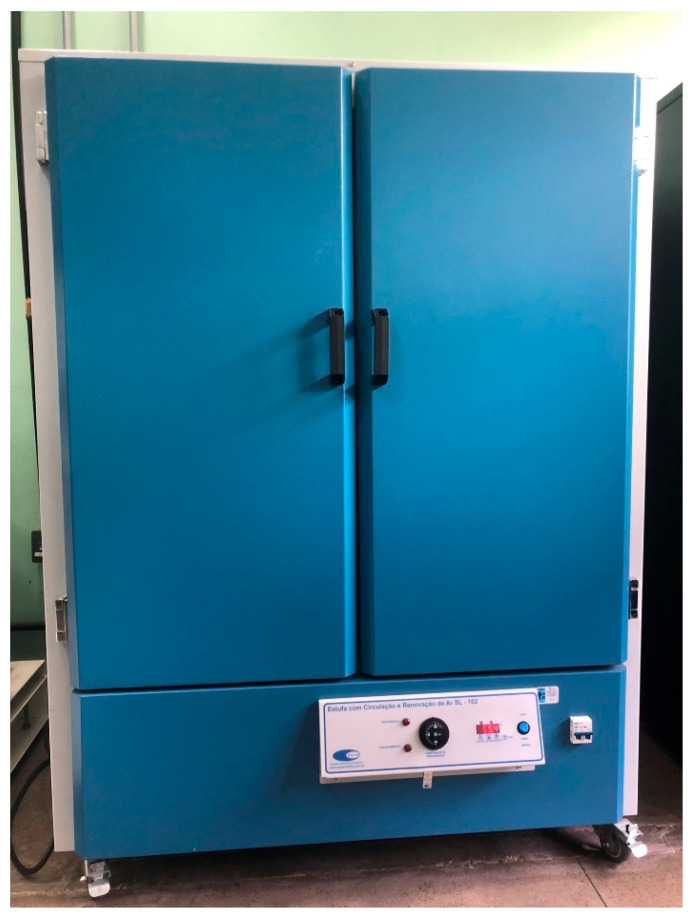
Greenhouse with circulation and air renewal SL—102.

Similar to Yang et al. (2019) [17], after the filament was granulated, it was placed in a controlled oven with circulation and renewal, and left in the oven for 8 h at 80 °C, as depicted in Figure 6. Since PLA is a hygroscopic material, the oven process is necessary to prevent moisture absorption and potential defects when obtaining the final composite. This process is fundamental, as hygroscopic materials like PLA can absorb moisture, leading to defects in the final composite, a common challenge in filament manufacturing for 3D printing.

Next, the process of preparing for the CNT was initiated, starting with the measurement of mass. The amount of CNT, which specifies a mass variation of 0~1% CNT relative to the PLA. An analytical balance, model L SERIES LA 110—ACCULAB, from the materials and processing laboratory at UEA, was used for measuring these masses.

**Figure 7:**
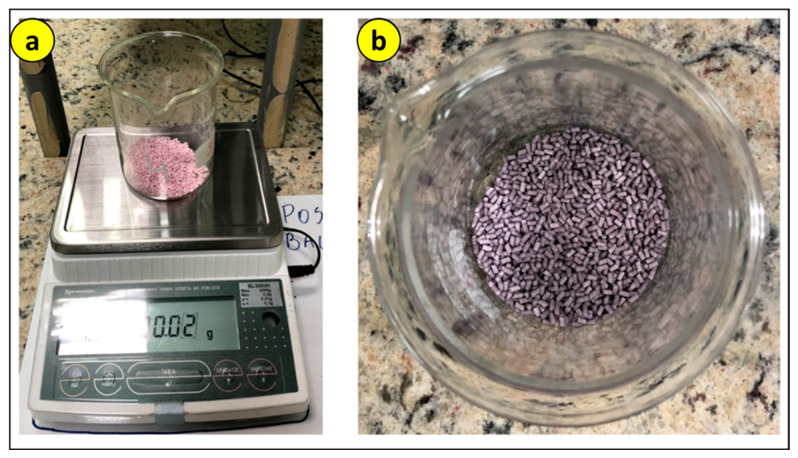
Mixing processes: (**a**) weighing of PLA; (**b**) PLA mixed with CNT.

After the 8 h oven period to keep the PLA moisture-free, the mixing process with the CNT was initiated. To ensure homogeneous dispersion, the PLA pellets and MWCNT powder were dry-mixed in a beaker for 15 min before being fed into the extruder. This pre-mixing step is crucial to prevent nanotube agglomeration, which could compromise the mechanical properties of the nanocomposite. Firstly, the mass of PLA was measured, and then, in the same breaker, the necessary amount of CNT was added according to Table 2. As depicted in Figure 7, the material exhibited a darker coloration compared to its initial color due to the mixture.

**Figure 8:**
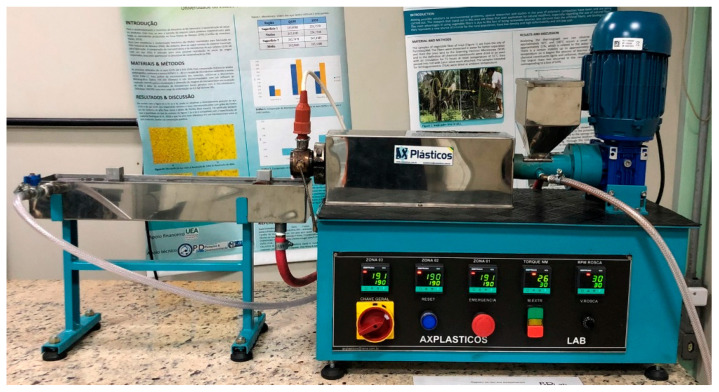
AX PLÁSTICOS mini-extruder.

The next step involved filament fabrication. For this purpose, a mini-extruder from AX Plásticos, as shown in Figure 8, was used. The extrusion process utilized the PLA-CNT mixture to generate the filament, with variations in RPM and extrusion temperature. Cooling was achieved using water at 28 °C.

**Figure 9:**
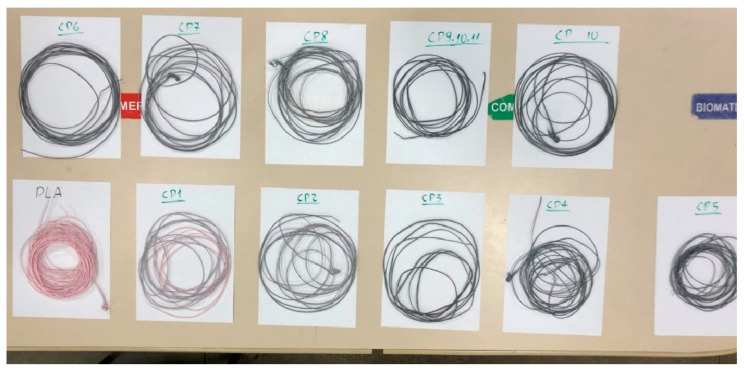
Manufactured filaments.

A total of 11 filaments were manufactured according to the characteristics outlined in Table 2. As depicted in Figure 9, the filaments exhibited different colors due to the varying amounts of CNT in each.

### 2.3. Manufacturing of Test Specimens

**Figure 10:**
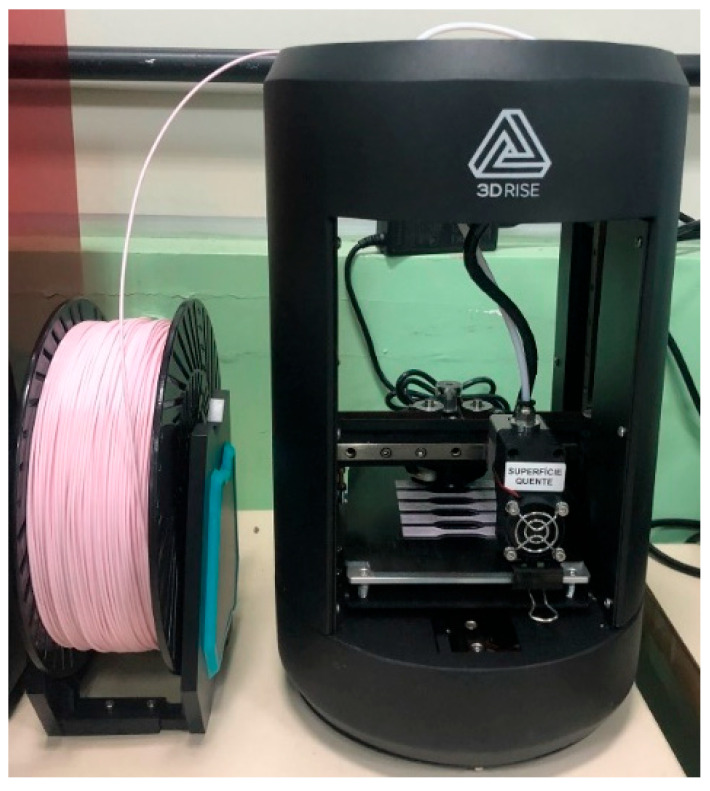
Printing of pure PLA test specimens.

With the filament fabrication completed, the next step was to obtain the test specimens. For this purpose, a 3D printer, brand 3D Rise FDM Mini, Brazil, as shown in Figure 10, was used.

Printer Characteristics:

- Printing Technology: Fused Deposition Modeling (FDM).
- Layer Resolution: 100 Microns.
- Print Volume: 100 × 140 × 120 mm.
- Precision: X and Y Axes: 11 Microns (0.0011 mm); Z Axis: 2.5 Microns (0.00025 mm).
- Filament Diameter: 1.75 mm.
- Compatible Filaments: PLA.

**Figure 11:**
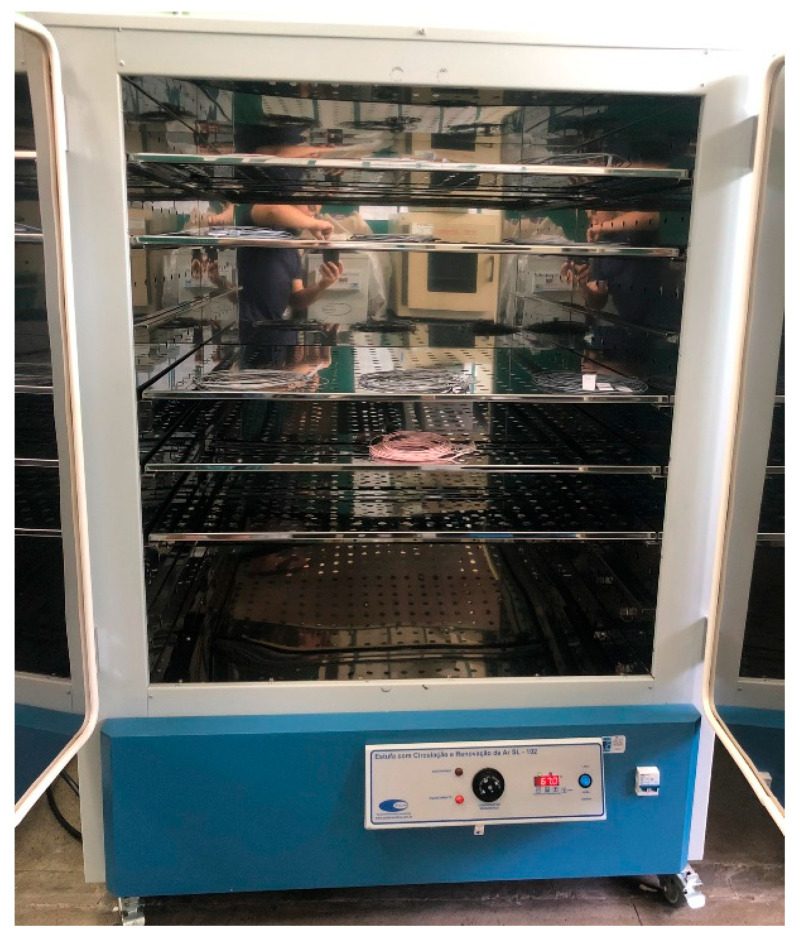
Filament oven process.

The acquired filaments were once again placed in the oven, as shown in Figure 11, as they were previously cooled in water. During this stage, the filaments remained in the oven for 6 h at a temperature of 60 °C.

The printing parameters were optimized through iterative preliminary trials to obtain parts without visible defects, such as delamination or underextrusion. The final parameters varied slightly with CNT content to compensate for changes in material viscosity. The printer was prepared and adjusted with the following parameters to accommodate the filaments:

- Print Quality: 0.2 mm.
- Print Speed: 50 mm/s.
- Outer Perimeter Speed: 45 mm/s.
- Infill Speed: 80 mm/s.
- Infill Density: 80 mm/s.
- Flow Rate: 110%.
- Extruder Nozzle Temperature: 210 °C.
- Infill: 40%.
- Print Orientation: 45°/−45°.

It is worth noting that other printing parameters were tested; however, they were unsuccessful, resulting in issues with infill and deformation. The careful selection of these parameters is crucial, as print orientation and infill density directly influence the anisotropy and magnitude of the mechanical strength of the parts [18]. Furthermore, challenges such as warping and bed adhesion are common, especially with polymers like HDPE, and require optimized strategies to ensure part quality [19].

**Figure 12:**
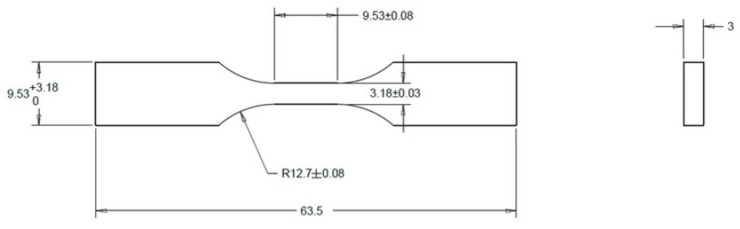
Test specimen according to ASTM D638-Type 5 (all measurements in mm).

With the parameters defined, the filaments were sequentially removed from the oven to manufacture the test specimens on the 3D printer. The test specimen standard used was according to ASTM D638-14 Type 5 [20], as shown in Figure 12.

**Figure 13:**
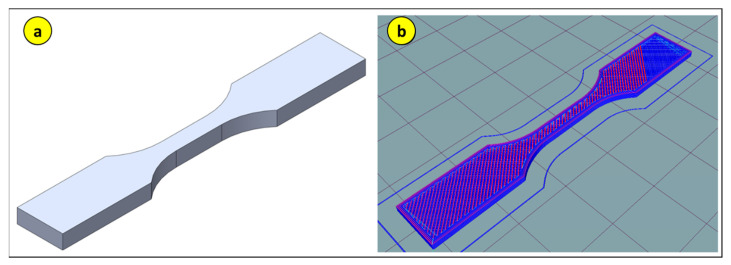
ASTM D638 Type 5: (**a**) test specimen modeling in SolidWorks; (**b**) slicing of the test specimen in Repetier-Host.

Before starting the printing process, the test specimen was modeled Dassault Systèmes. *SOLIDWORKS 2019 SP4* [Cad software]. Vélizy-Villacoublay, França: Dassault Systèmes., as shown in Figure 13. Subsequently, it was exported to Hot-World GmbH & Co. KG. *Repetier-Host 2.3.1* [Computer software]. Willich, Alemanha: Hot-World GmbH & Co. KG for slicing and printing.

**Figure 14:**
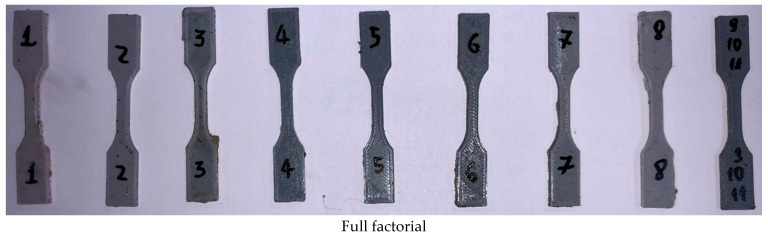

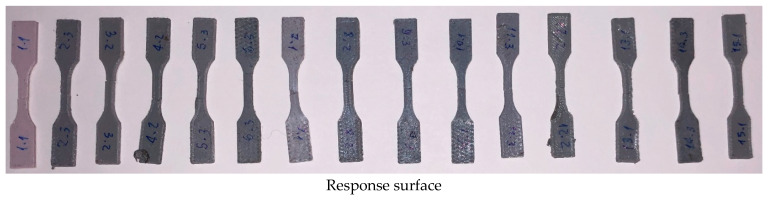
Acquired test specimens.

The next step was the printing of the test specimens. For the full factorial cases, 11 specimens were printed, and for the response surface, 15 specimens were printed, as depicted in Figure 14.

For the printing process, there were some difficulties requiring a long time and many attempts for the fabrication of the test specimens, such as

- Adjusting the extruder nozzle temperature;
- Extruder nozzle clogging;
- Print speed;
- Ambient temperature.

### 2.4. Thermogravimetric Analysis (TGA) Methdology

The analysis was conducted on the nanocomposites that exhibited the best mechanical results, aiming to determine the onset degradation temperature and characterize the composition based on the mass losses of each thermal event.

Thermogravimetric analysis (TGA) was conducted using a DTG-60 apparatus from SHIMADZU, located in the Laboratory of Electrochemistry and Energy (LEEN) at FT/UFAM. Approximately 20 mg of the nanocomposite samples was used, with a heating rate of 10 °C/min up to a final temperature of 800 °C, under a nitrogen gas flow N2 (50 mL/min). The crucible used in this analysis was a 90-microliter uncovered alumina crucible.

### 2.5. Scanning Electron Microscopy (SEM) Methdology

The images of the fracture surfaces of the nanocomposites (obtained from the tensile tests) were captured using a scanning electron microscope (SEM). They were coated with a thin layer of cathodically sputtered silver on the samples. The equipment used was the SEM at the Multi-User Center for Biomedical Phenomena Analysis (CMABio) at the University of the State of Amazonas (UEA). It featured a tungsten capillary filament (negative electrode) with an applied potential difference of 5.0 kV, using the Jeol JSM-IT500HR model.

### 2.6. Transmission Electron Microscopy (TEM) Methdology

Images of the CNTs were obtained to verify their dimensions and predict their behavior. Additionally, images of the nanocomposites were captured to observe the behavior of the nanoparticles with PLA. The nanocomposites characterized by transmission electron microscopy (TEM) were prepared from injected filaments subsequently milled. The samples were produced through ultra-thin cuts (~100 nm), using diamond knives in a Leica UC7 ultramicrotome.

The micrographs were obtained using a transmission electron microscope with a field emission electron gun operating at 80 kV, with the JEOL JEM-1400Flash equipment, located at the Multi-User Center for Biomedical Phenomena Analysis (CMABio) at the University of the State of Amazonas (UEA).

### 2.7. Fourier Transform Infrared (FTIR) Radiation

In order to analyze the evidence of the presence of functional groups present in the structure of the nanocomposite and pure PLA, analysis was carried out via FTIR.

In the following study, FTIR analysis was performed on the nanocomposites with the best mechanical results. The spectra were obtained using a Thermo Electron spectrometer (Model Nicoleti S10) located in the infrared spectroscopy laboratory at the analytical center of UFAM, with 32 scans in the range of 4000 to 400 cm^−1^ and a resolution of 4 cm^−1^. Each sample was mixed with KBr in a 1:100 ratio and pressed into a disc at a pressure of 80 kN for 2 min.

### 2.8. Tensile Mechanical Testing

The tensile test was conducted according to the ASTM D638 standard, [21] utilizing the INSTRON universal testing machine, type 5984, located in the R&D laboratory of the School of Technology at the University of the State of Amazonas (EST-UEA). The load cell capacity was 150 kN, and the Bluehill 3 software was used. The tests were conducted at room temperature.

Through this test, the mechanical properties of the printed material, such as tensile strength, were determined, to be inserted into our worksheet as output data.

**Figure 15:**
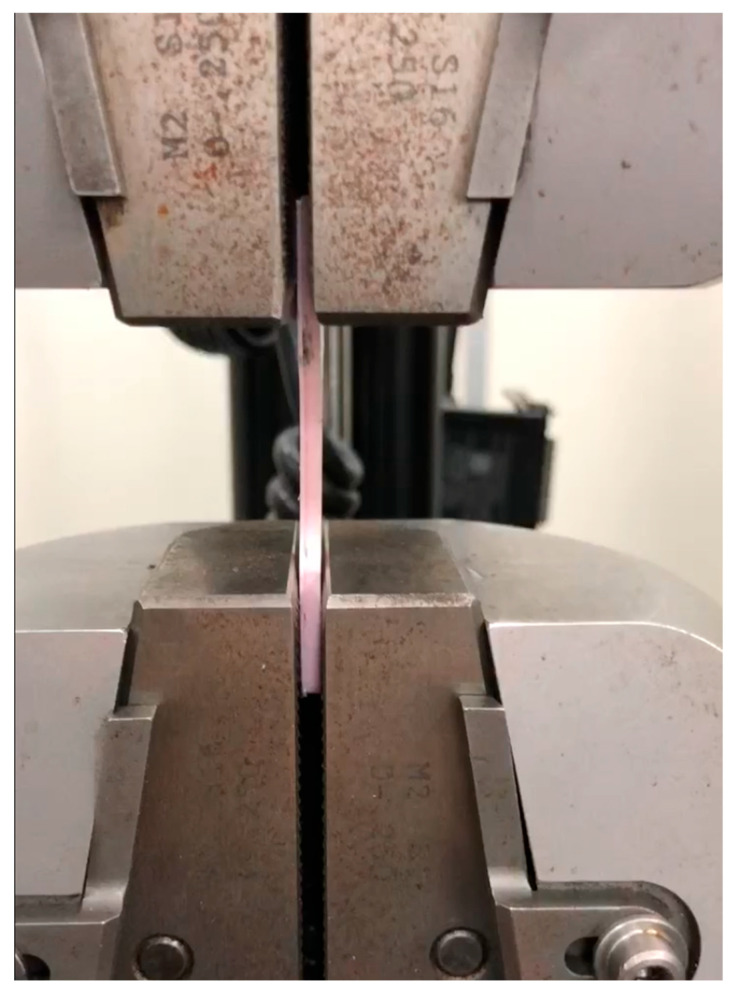
Tensile test.

For this purpose, a tensile test was conducted using the Instron equipment, following the ASTM 638 standard, as shown in Figure 15.

### 2.9. Computer Simulation

For the computational numerical simulation, finite element analysis was conducted using the optimized material data, composed of PLA and CNT, to perform a comparison of the properties applied in the use of the material in the propeller, as proposed in this study.

For the PLA + CNT nanocomposite, the properties found in the Ansys Discovery library, such as the stress–strain curve, were inputted, as shown in Table 4.

Once all the necessary material information had been inputted into the program for finite element analysis, in this case, the analysis of propeller utilization, the calculation application, the program’s solver, was initiated. As the focus of this work was on developing an alternative material for propeller manufacturing and was interested in material failure as its applicability limit, the von Mises failure criterion was used as the upper limit. Although the Tresca criterion provides a reasonable assumption for yielding in ductile materials, the von Mises theory correlates better with experimental data and, therefore, is generally more commonly used.

With the material defined, the next step was to define the simulation characteristics of the propeller to make the situation as realistic as possible, using the following values and conditions.

- Gravity: 9.81 m/s^2^.
- Propeller rotation: 12,000 rpm.
- Pressure on the blade surface from the bottom: 5.9 MPa.
- Load on the propeller: 19.6 N.

The mentioned values were calculated based on the actual weight of the drone, its acceleration, and maximum ascent and descent speeds [1].

The necessary options for preprocessing are listed in the order in which parameters should be entered. It starts with defining the material, and with that, all material properties are used to form the stiffness matrix. Once the material is confirmed (i), you move on to the second option, defining the use of gravity properties (ii). Next is defining the propeller speed (iii) and its respective connections (iv), which are the components in contact, especially in systems or assembly designs, where you want to understand not only the individual behavior of a component but also the result of its interaction with other components, such as the shaft connected to the propeller.

**Figure 16:**
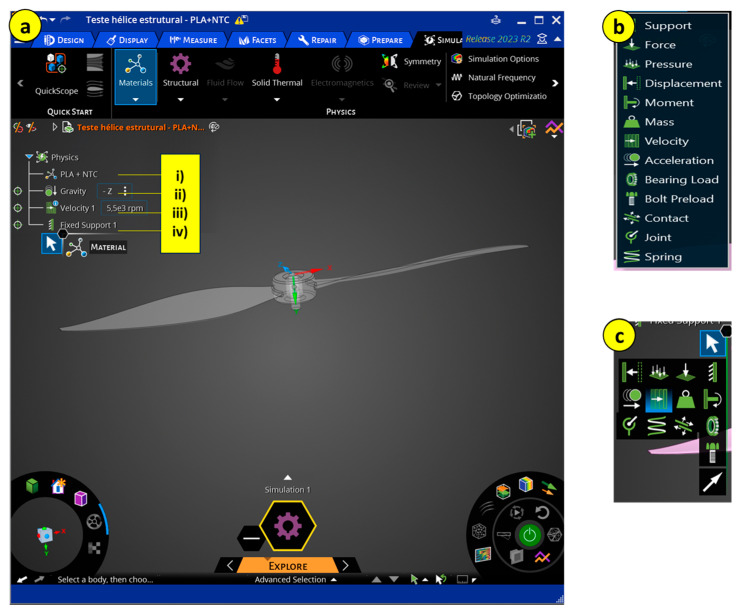
Simulation: (**a**) Ansys Discovery interface; (**b**) boundary conditions; (**c**) option to apply values.

The simulation was performed using the Ansys Discovery software, student version 2023, focusing solely on structural simulation (Figure 16).

The manufacturing of the optimized nanocomposite aimed to enhance mechanical characteristics. This involved finding the best possible combination of PLA and CNT, which was determined using Design of Experiments (DOE) with a full factorial design and response surface analysis, both conducted using Minitab software.

By varying the percentage of CNT, temperature, and rotation, a filament with elevated properties for use in FFF (Fused Filament Fabrication) 3D printers was developed, expanding the utility of PLA in various applications.

## 3. Discussion

### 3.1. Full Factorial

After conducting the tensile tests, in which three replicates were used for filament configuration, totaling 33 samples, the full factorial model was utilized to obtain information on what truly matters for the process, as shown in Table 5.

**Figure 17:**
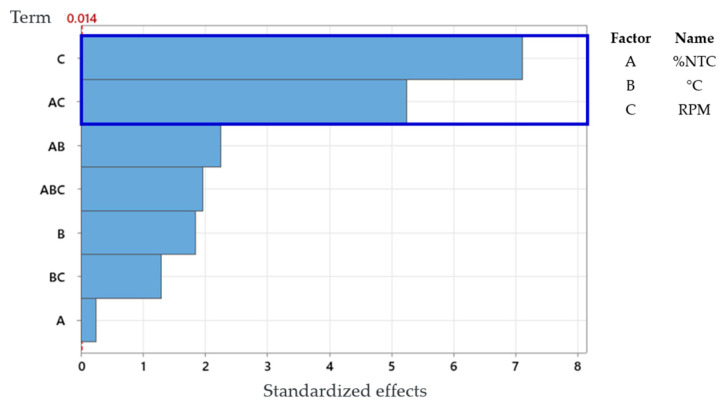
Pareto chart of standardized effects (the response is tensile strength; α = 0.99).

Through the Pareto chart, Figure 17, we can identify the factors that have the most impact on the end of the process. In the case of the graph below, it can be observed that factors C (RPM) and the interaction AC (RPM and %CNT) significantly influence the process.

**Figure 18:**
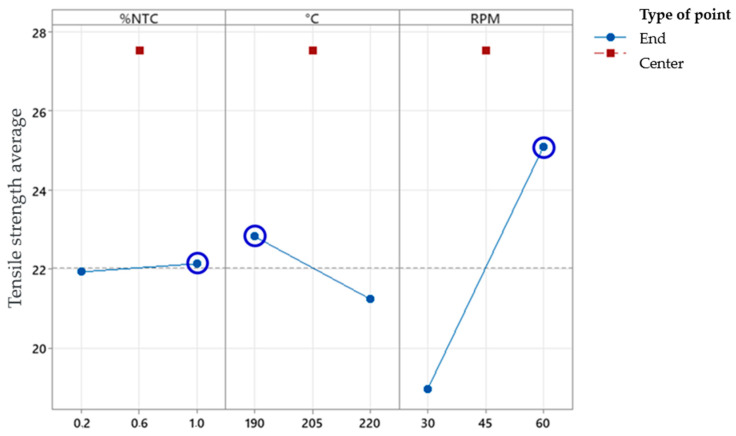
Main effects plot for tensile strength—adjusted means.

The main effects plot, Figure 18, evaluated the configuration of isolated variables that had the most impact on the process individually. The steeper the slope of the line, the greater the relevance of the variables and their levels in the final result.

In the analyzed graph, Figure 18, it is observed that the slope of the line corresponding to the variable “RPM” is greater than the others, indicating a higher impact on the final tensile strength values.

**Figure 19:**
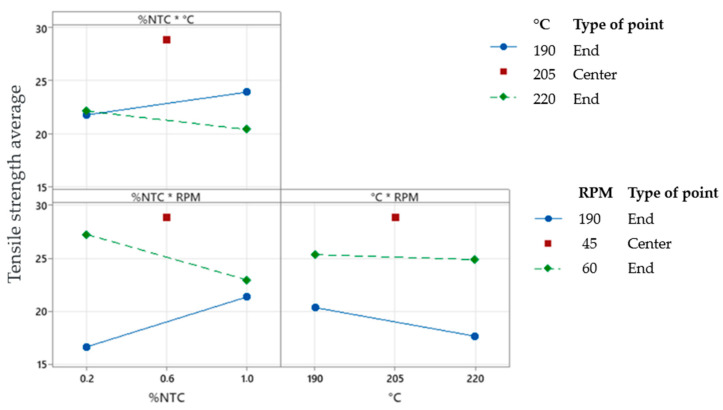
Interaction plot for tensile strength. (*) Units.

The interaction plot, Figure 19, assessed the interactions between variables and their impact on the process. The less parallel the lines are to each other, the greater the interaction between the variables. When there is a crossing, there is interaction. In the obtained graph, the interaction between “%CNT” and “RPM”, and between “%CNT” and “°C”, was evaluated.

For the study using the full factorial design, it can be concluded that the factor “RPM” has significant importance in the process, as shown by the Pareto diagram and the main effects plot, as well as the interaction between “%CNT” and “RPM”, and between “%CNT” and “°C”.

### 3.2. Response Surface

After conducting the new tensile tests, in which three replicates were used for filament configuration, totaling 45 samples, the response was included in the response surface model to obtain information on how the terms affect the response, as shown in Table 6.

**Figure 20:**
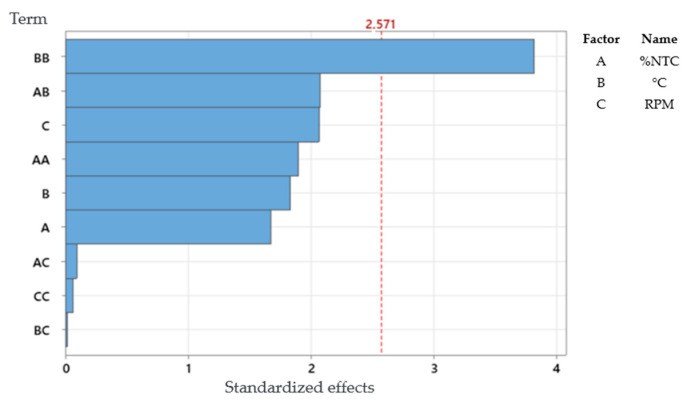
Pareto chart of standardized effects (the response is stress; α = 0.05).

If we compare the Pareto chart of the response surface, Figure 20, with the Pareto chart of the full factorial design, Figure 17, we can observe that there are more combinations. This is because in the response surface experiment, the quadratic form of all factors is considered. Thus, we can conclude that combination BB stands out in relation to the others, meaning it significantly affects the response. Additionally, even below the critical line, combinations AB and C also significantly affect the response.

**Figure 21:**
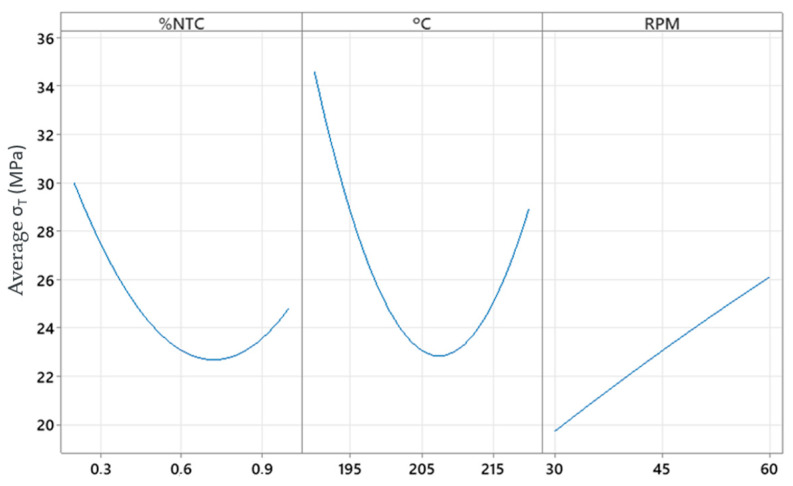
Main effects plot for stress.

In the main effects plot, Figure 21, we can see the actual behavior of the factors on the response, noting that they are not linear models but quadratic for %CNT and °C, meaning there is a region where optimized values can be found within a working range.

**Figure 22:**
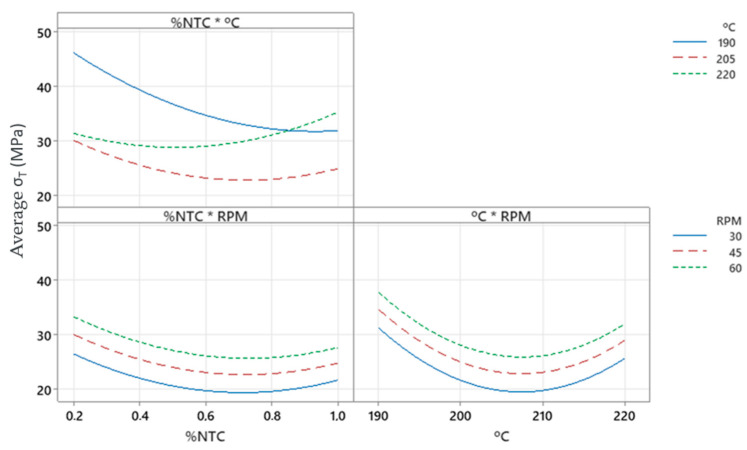
Interaction plot for stress. (*): Units.

In the interaction plot, Figure 22, we can observe that we have higher stress values for 190 °C, 60 RPM, and 0.2% CNT.

**Figure 23:**
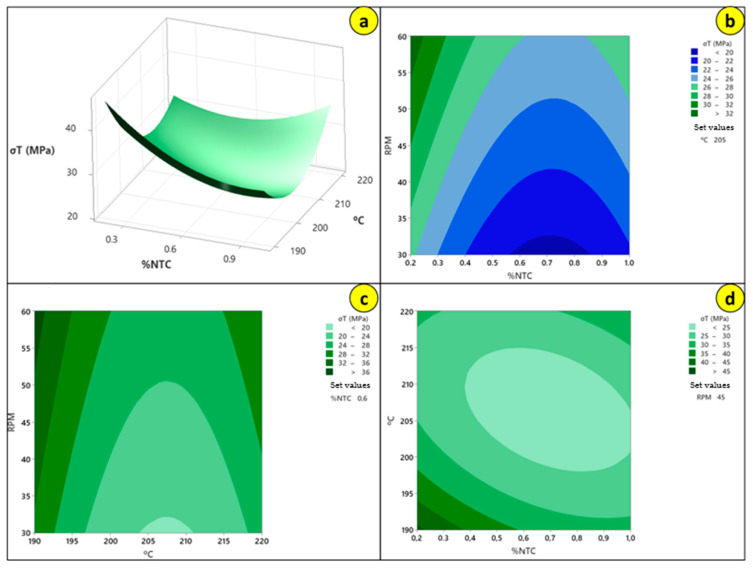
Factors behavior: (**a**) stress surface plot versus temperature and %CNT; (**b**) stress contour plot versus RPM and %CNT; (**c**) stress contour plot versus RPM and temperature; (**d**) stress contour plot versus temperature and %CNT.

In Figure 23, we can observe the behavior of the factors alongside the response, allowing us to identify the working region as previously discussed. Thus, we can find the optimized filament, which according to the graphs is the combination of 190 °C, 60 RPM, and 0.2% CNT, but similar results may be found in the highlighted regions.

**Figure 24:**
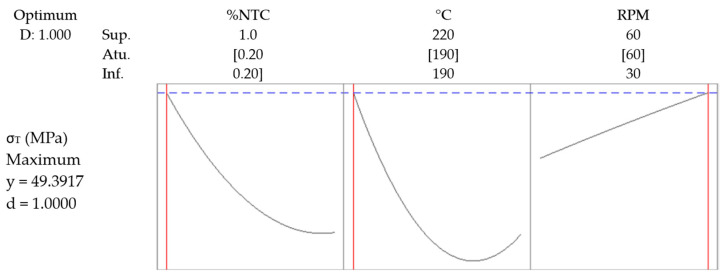
Response optimization. [Concentration, %NTC], [Temperature, °C], [Rotation, RPM].

To confirm that the values analyzed in the graphs were consistent, an optimization was conducted to identify the maximized parameters and the optimized value. The optimized response can be found in Figure 24.

Through the analysis conducted, it was observed that not only the concentration of nanoparticles can influence the mechanical characteristics of the material, but also the temperature and the rotation at which this material is extruded. It can be seen that for each combination of factors, there is a different result, with some combinations yielding higher mechanical results without the need for high concentrations of nanoparticles.

For the work conducted, it was observed that the best result is achieved with the parameters of 190 °C, 60 RPM, and 0.2% CNT. However, according to Figure 23, elevated values can be found for other regions, allowing for the analysis of new parameters and new results depending on the research objectives. Through the interaction of these factors, good values can be found within the ranges:

- 55 to 60 RPM (Figure 23b);
- 192 to 190 °C and >220 °C (Figure 23c);
- 0.6 to 0.2%CNT (Figure 23d).

Traversing the surface of Figure 7a, high stress values can be found.

Through this research, it can be confirmed that the Design of Experiments (DOE) tool significantly contributes to determining the best configuration that a set of controllable variables should have to maximize a desired outcome.

From the response surface analysis (RSM) performed in Minitab software, a regression equation was generated that describes the relationship between tensile strength (σT) and the studied factors:

- CNT content (A);
- Temperature (B);
- RPM (C).

The equation, in terms of uncoded factors, allows for the prediction of the material’s strength within the investigated experimental range:

$$\sigma_{T}\left(MPa\right)=1780-192.9*A-16.49*B+0.33*C+27.1*A^{2}+0.758*A*B-0.035*A*C+0.0387*B^{2}-0.0002*B*C-0.0006*C^{2}$$

This quadratic equation captures both the linear effects of each factor and the interactions between them, as well as the quadratic effects (curvature), which were shown to be significant, as indicated by the Pareto chart (Figure 20). The model presented an R^2^ of 86.53%, indicating a good fit to the experimental data and confirming its utility for process optimization.

### 3.3. Mechanical Test Comparison

**Figure 25:**
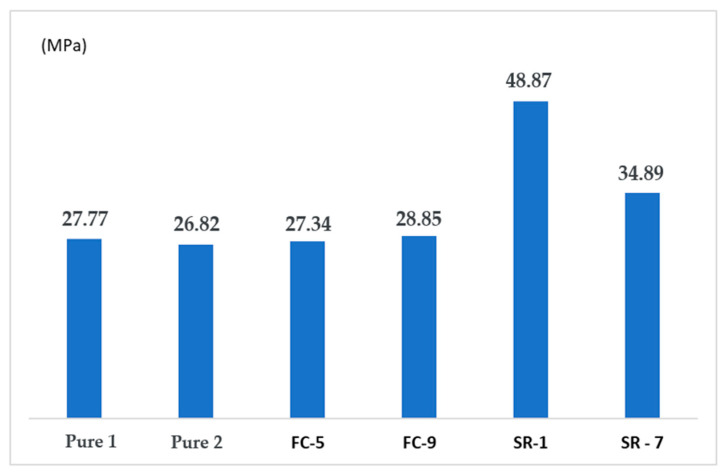
Comparative graph of tensile stress (MPa) between Pure Material x FC x SR.

When comparing the values obtained at each stage, the variation in stresses can be clearly seen, as shown in Figure 25. Comparing the values of the pure material with those obtained in the response surface, we observe a gain of 43% compared to CP SR-1 and 20% compared to CP SR-7.

**Figure 26:**
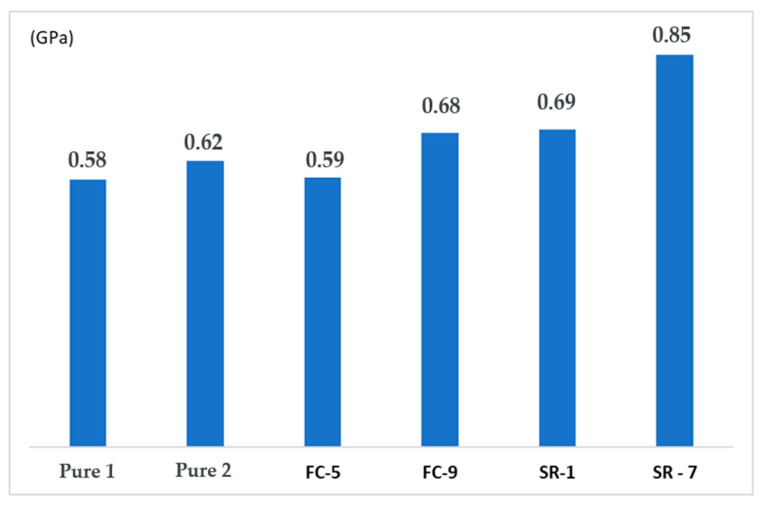
Comparative chart of modulus of elasticity (MPa) between Pure x FC x SR material.

The same applies to the modulus of elasticity, as seen in Figure 26, where there is an increase due to the nanotubes and the interaction between temperature and screw rotation.

According to [22], with the addition of carbon nanotubes to the composite, the material was heterogeneously nucleated, increasing the crystallization speed, temperature, and degree of crystallinity. This effect contributes to a gain in the modulus of elasticity because it reduces the mobility of the matrix chains. This behavior explains why the modulus of elasticity of pure PLA was lower than that of the nanocomposite obtained after the addition of carbon nanotubes. This behavior is consistent with other studies showing that reinforcements like nanotubes or fibers increase the composite’s stiffness by restricting the mobility of the polymer chains [23].

The variation in extrusion and printing temperatures, as well as the increase in these parameters (temperature, %CNT, and rotation), reduces the molding process cycle time. This is because it reduces the critical free energy for core consolidation and accelerates the overall crystallization process.

In the research conducted by [17], it was reported that the addition of low concentrations of CNT (1 and 3% by weight) in PLA matrices increases the degree of crystallinity and leads to a beneficial effect on the tensile strength and fusion stability of PLA components, facilitating the 3D printing process. Therefore, it is feasible to evaluate lower concentrations of CNT in PLA matrices while maintaining the desired improvements in mechanical component performance. Similarly, studies with recycled glass fibers in polypropylene matrices have shown that increasing the fiber content improves tensile strength up to an optimal point, after which fiber agglomeration can lead to a reduction in performance [24].

In Figure 25, the samples “SR-1” and “SR-7” represent two of the experimental configurations from the response surface design (Table 6). The specific details are as follows:

- SR-1 (Run 11): Corresponds to the sample that exhibited the highest tensile strength (48.9 MPa), manufactured with 0.2% CNT, at a temperature of 190 °C and 45 RPM **.
- SR-7 (Run 15): Showed a strength of 34.9 MPa, manufactured with 0.2% CNT, at 205 °C and 60 RPM **.

The superiority of sample SR-1 can be attributed to the optimized combination of processing parameters. The response surface contour plots (Figure 23) show that the region of maximum tensile strength is found at lower temperatures (near 190 °C) and intermediate to high rotations (45–60 RPM). The temperature of 190 °C (used in SR-1) is ideal as it ensures proper melting of the PLA without initiating thermal degradation, which can occur at higher temperatures like 205 °C (used in SR-7), weakening the polymer matrix. Additionally, the rotation of 45 RPM in SR-1 appears to have provided sufficient shear for good CNT dispersion without excessively damaging the polymer chains. In contrast, although SR-7 used a higher rotation (60 RPM), the higher temperature (205 °C) likely had a predominant deleterious effect, resulting in lower mechanical strength. Therefore, the higher strength of SR-1 is a result of a favorable synergy between a lower processing temperature and an adequate rotation, which maximized the matrix integrity and the dispersion of the nanoreinforcement.

### 3.4. Fracture Study by Scanning Electron Microscopy (SEM)

**Figure 27:**
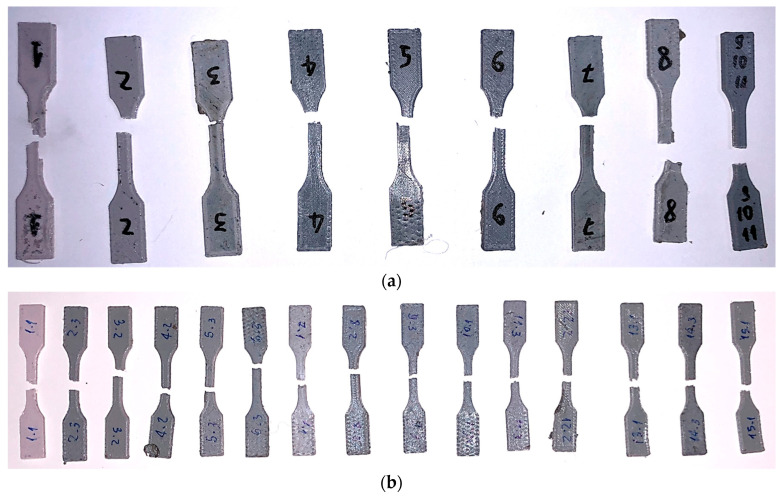
Specimens after tensile test fracture. The numbers refer to the design shows in Table 5 and Table 6, where: (**a**) For the full factorial design with range 1 to 9 and (**b**) The response surface design with range 1 to 15.

The tensile test was conducted for all specimens as planned in the worksheet. In Figure 27, we can see that the zone of fracture in some cases (6) was far from the central region, possibly due to a failure during the printing process, which may contribute to the weakening of the region. However, in the majority (18), fracture occurred in the central region.

To demonstrate the behavior of the samples regarding the proportions of CNT in the PLA matrix, and how the material processing influenced the properties, a systematic analysis of their morphologies was conducted.

The test specimens were printed with 40% infill, and regions of non-filled areas can be observed in the cross-sections of the samples, leaving a kind of shell. However, there is excellent cohesion between the deposited filaments, which directly contributes to the performance of the samples in mechanical analyses.

**Figure 28:**
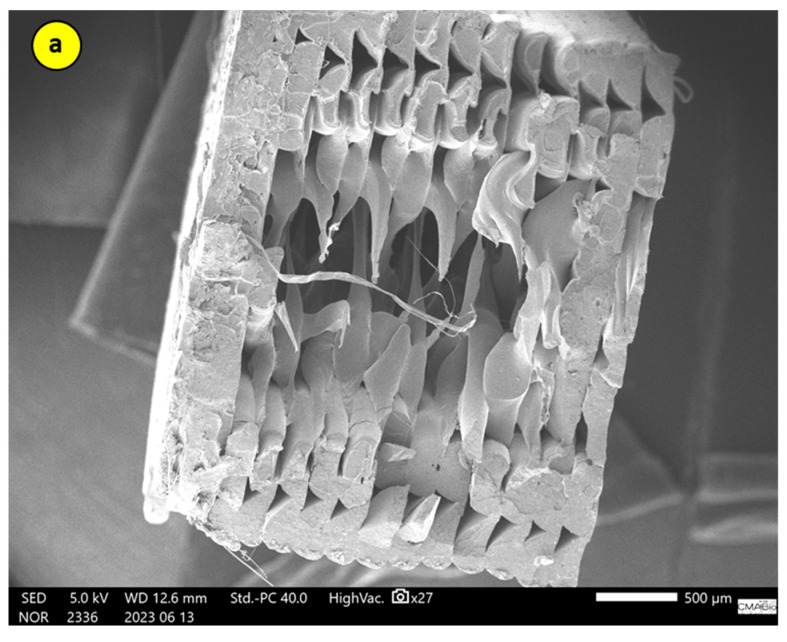

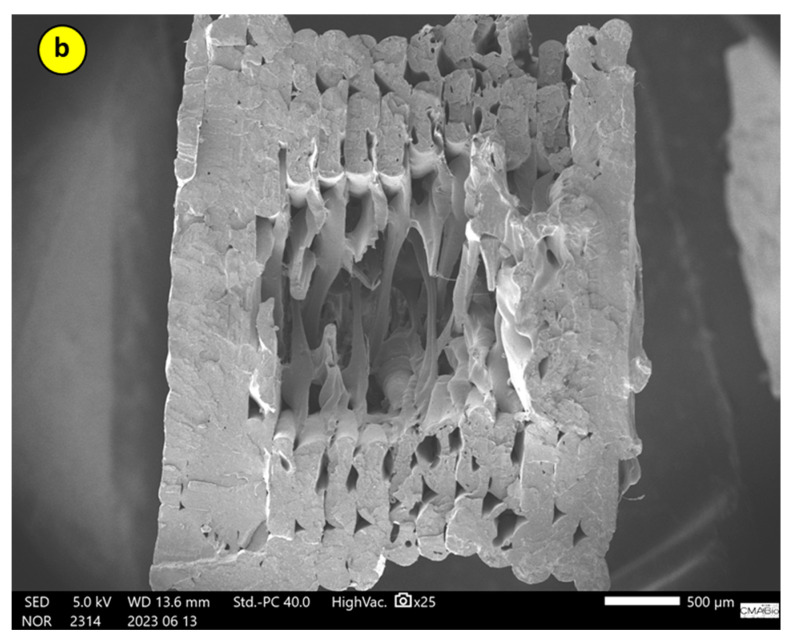
SEM micrographs of the fracture surfaces of the specimens corresponding to a-SR-1 and b-SR-7 from the response surface (5 kV, 500 µm).

**Figure 29:**
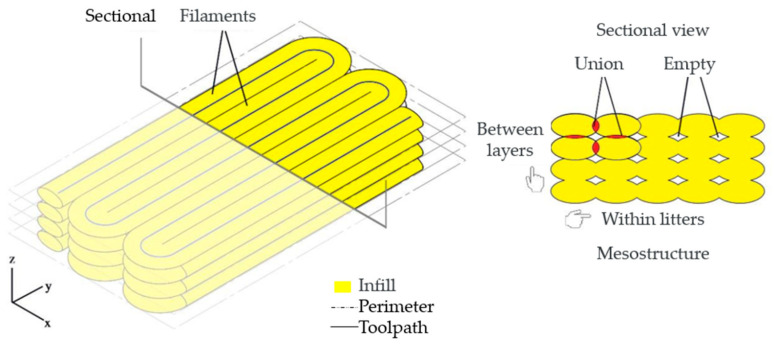
Similar scheme of the specimens corresponding to SR-1 and SR-7 from the response surface.

As per [18], parts manufactured by the 3D printing process via extrusion exhibit a mesostructure formed by a density of voids and the existence of filament-to-filament bonds deposited within and between the layers, as shown in Figure 28 and Figure 29.

The mesostructure, depicted in Figure 28 and Figure 29, is determined by the trajectories of filament deposition and process parameters. The presence of voids inside the parts is due to the oblong shape of the filaments that compose them. The size and shape of the voids, as well as the extent of the bond between individual filaments (contact area), are factors that influence the anisotropy and magnitude of the mechanical strength developed by the parts [17,18].

**Figure 30:**
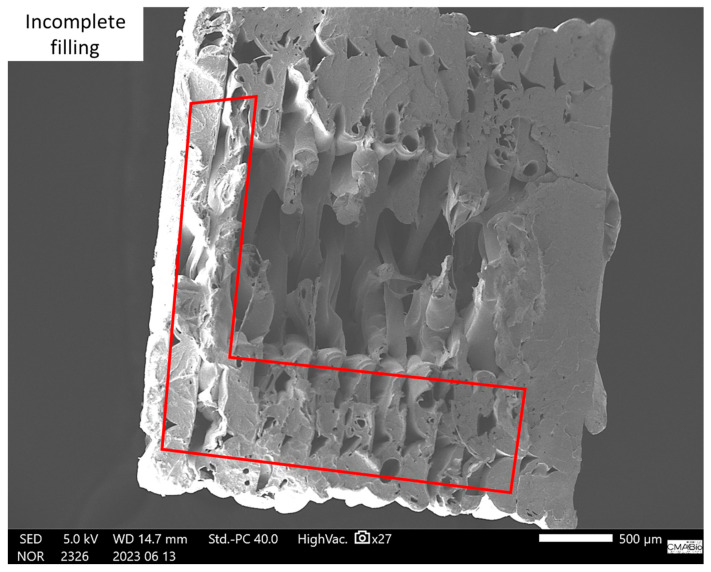
SEM micrograph of the fracture surface of the specimen corresponding to SR-11 from the response surface (5 kV, 500 µm).

When comparing Figure 28 for SR-1 and SR-7, which have a concentration of 0.2% CNT, to Figure 30 for SR-11, we can see a slight change. In the latter figure, we start to see some printing defects, resulting in pores, which can influence mechanical tests. This was observed during the tests, where SR-1 and SR-7 exhibited higher stresses than SR-11. This indicates that increasing the amount of CNT reduces the efficiency of material cohesion during the printing process.

Although the manuscript identified anisotropy (evidenced by the voids), we did not evaluate the impact of this phenomenon on the performance of the composite itself. To better understand this anisotropy, multidirectional mechanical tests could be performed—for example, tensile and/or flexural tests in different orientations (e.g., X-Y and Z)—to quantify the anisotropy and understand the material’s true strength.

Building on this, the direct implications of anisotropy on the service life and safety of the propellers could be studied in future work, as interlayer failure can lead to catastrophic failure in service. In summary, we acknowledge the absence of a dedicated analysis of anisotropy itself. We understand that merely observing voids is not sufficient to fully characterize the complete mechanical behavior of an FDM-printed part, as other parameters are also important.

The qualitative analysis of the TEM/SEM micrographs (Figure 28, Figure 29 and Figure 30) demonstrates the presence of carbon nanotube (CNT) agglomerates, confirming one of the main challenges in producing high-performance polymer nanocomposites: ensuring the proper distribution and dispersion of the CNTs.

The absence of quantitative metrics, such as agglomerate size distribution, makes the link between dispersion quality and mechanical performance speculative. This is because CNT agglomerates act as stress concentrators, creating regions of weakness in the material and consequently degrading its mechanical properties.

Poor dispersion results in a less homogeneous material, which can lead to the formation of voids and filling defects in printed parts, affecting their quality and performance. A good dispersion provides a larger available surface area to interact with the polymer matrix, maximizing the filler’s influence on the material’s properties. These observations are consistent with studies by [25,26,27].

Although the micrographs do not provide quantitative data, they offer visual evidence that corroborates the importance of dispersion for performance. The presence of agglomerates in the micrographs, along with the observation that an increased amount of CNTs can reduce the material’s cohesion during printing, justifies why the material processing influenced its properties. These considerations are also reported in the literature, such as in [28].

Alternatively, the TEM analysis in the document confirmed the presence of CNTs, their cylindrical shape, and a good distribution within the matrix for materials with a lower percentage of CNTs. However, the same result was not achieved with higher concentrations, possibly due to the printer’s characteristics. Raman analysis would be quite interesting; however, due to infrastructural limitations, we do not currently have access to this characterization method, not even through partner laboratories. Furthermore, in national laboratories where such characterizations are possible, the wait time is over 90 days. We do plan to perform this analysis, but in future work, once this line of research is more established.

Ref. [26] explains that ensuring proper distribution and dispersion of CNT remains one of the biggest challenges for the production of high-performance polymeric nanocomposites. Good dispersion provides a larger surface area available to interact with the polymer matrix, maximizing the influence of the filler on the material properties. Additionally, CNT clusters can act as stress concentrators, creating brittle regions and reducing the mechanical properties of the material. This is a central challenge in manufacturing polymer nanocomposites, as good dispersion maximizes the available surface area for interaction with the matrix, while agglomerates act as stress concentrators, impairing mechanical properties [27].

These same clusters, according to [18], can cause flow interruptions during the printing process. Poor distribution and dispersion of CNT in the PLA matrix result in less homogeneous material with different viscosity regions, which can cause voids and fill defects in the printed parts, jeopardizing the quality of the printed part. Poor dispersion not only affects mechanical properties but can also cause flow interruptions during the printing process, resulting in voids and filling defects in the printed parts [24].

### 3.5. Transmission Electron Microscopy (TEM)

**Figure 31:**
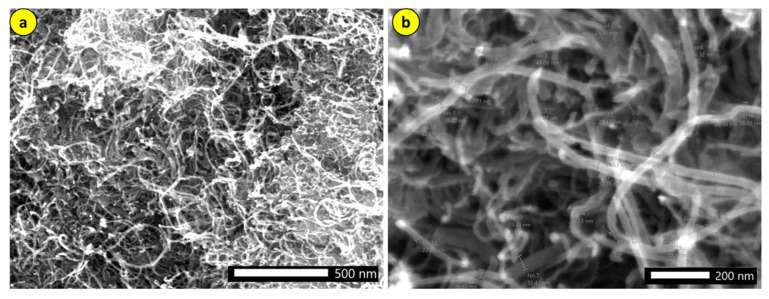
SEM of the nanotubes used: (**a**) 500 μm; (**b**) 100 nm.

First, TEM analysis was performed on the CNT to verify its dimensions, showing an average diameter of 30 nm (see Figure 31).

For performing the TEM of the CPs, as well as the SEM, the samples with the best mechanical performance were used, as this is the main goal of this work.

**Figure 32:**
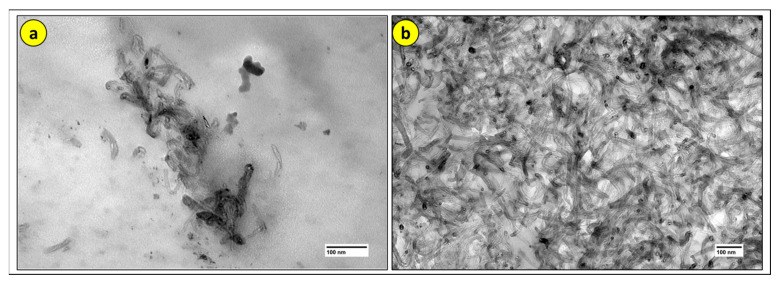
TEM image of the PLA/CNT nanocomposite: (**a**) CP 11; (**b**) CP1.

In the micrographs obtained from the material and analyzed by TEM, carbon nanotubes on the surface are observed, appearing as cylindrical shapes with small spherical tubes, as depicted in Figure 32. The pattern of particle size distribution and density appears random but clustered.

According to [21], Figure 32 illustrates the darker and lighter regions denoting areas of high and low concentrations of CNT distributed throughout the polymer matrix. Additionally, small tubes with different orientations formed by graphene sheets are observed within the matrix, which characterizes the behavior of CNT present in the PLA/CNT nanocomposite. Homogeneous dispersion is fundamental for the reinforcement to effectively contribute to the composite’s properties. In piezoresistive sensors, for example, the distribution of CNTs is what allows the formation of a conductive network sensitive to deformation [23].

Figure 32 clearly illustrates what has been discussed in the previous topic, that nanoparticle agglomerates and their poor dispersion can result in a less homogeneous material and can act as stress concentrators. This creates weaker regions and reduces the mechanical properties of the material.

While Figure 32a shows poor dispersion and CNT agglomerates, Figure 32b exhibits excellent dispersion with minimal agglomeration, justifying the values obtained in the properties, where CP 1 showed better properties than CP 11.

### 3.6. Thermogravimetric Analysis (TGA)

Just like for MET and MEV, the samples with the best mechanical performance were used, as this is the main objective of this study.

**Figure 33:**
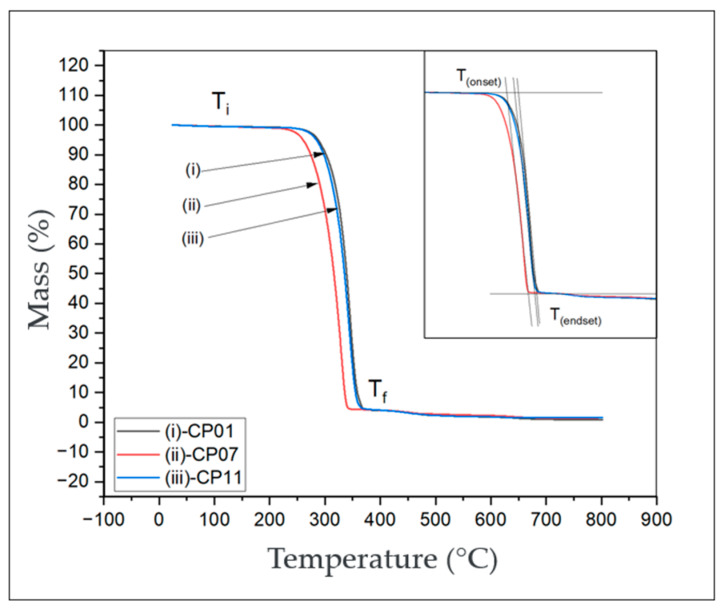
Thermogravimetric analysis graph comparing the test specimens.

The TGA technique was used to evaluate the thermal stability and decomposition of PLA with CNT (Figure 33 and Table 7). Results were obtained based on the mass loss as a function of temperature variation.

Figure 33 depicts a comparison between thermogravimetric analysis curves of test specimens CP01, CP07, and CP11, obtained through the worksheet used in the response surface, where it was possible to obtain the best stress values. Four regions with their changes in curvature of the curves are delimited in the graph, namely

- T_i_—The initial temperature is the lowest temperature at which the onset of mass change can be detected for a given set of experimental conditions.
- T_f_—The final temperature is the lowest temperature indicating that the process responsible for the mass change has been completed.
- T_onset_—It is identified by the crossing of the lines and is referred to as the extrapolated onset or mathematical onset.
- T_endset_—It is identified by the crossing of the lines and is referred to as the extrapolated end or mathematical end.

It is possible to visualize the four temperatures where two visibly altered areas intersect on the curves. At T_onset_, the onset of the maximum degradation rate is highlighted; for CPs 01, 07, and 11, respectively, this was 316.7 °C, 286.5 °C, and 307.7 °C, as shown in Table 7.

For CP 01, there was a mass loss of 92.5% until reaching the temperature of 363 °C. For CP 07, the mass loss was 95.7%, and for CP 11, it was 94.58%, all at the same temperature of 363 °C.

It is observed that in the T_f_ curve, the values of losses have minimal differences; for CPs 01, 07, and 11, respectively, these were 95.94%, 95.83%, and 95.79%, which could be due to the low concentration of carbon nanotubes.

From the moment the curve reaches the stable plateau of mass loss versus temperature, approximately at 638 °C, it is observed that the percentage of mass residue among the test specimens is minimal; for CPs 01, 07, and 11, respectively, this was 1.07%, 1.55%, and 1.54%. This occurs due to the low quantity of carbon nanotubes present in the nanocomposite. It is noted that the degradation temperature of the carbon nanotube (CNT) load is extremely high. In Table 8, it is possible to verify the percentages of mass losses and residues.

By adding carbon nanotubes (CNTs) in a considerable dispersion, it allows for an increase in the thermal stability of the material, as one would expect an improvement in the initial decomposition temperatures of the hybrid, since the carbon nanotubes (CNTs) act as insulators, serving as a barrier for the volatile products generated during decomposition [28].

### 3.7. Derivative Thermogravimetric Analysis (DTG)

**Figure 34:**
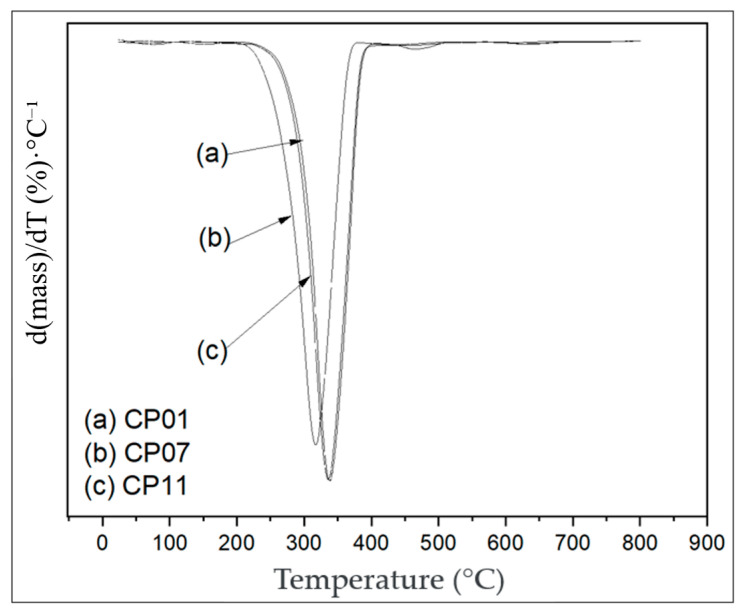
Derivative thermogravimetric analysis (DTG) graph.

Table 9, taken from Figure 34, shows the peaks and temperature differences among the test specimens.

The thermogravimetric analysis (TGA) curves are shown in Figure 33 for PLA and PLA/CNT. The initial degradation temperature of the materials was above 285 °C, with a mass loss exceeding 80%. The final degradation process was completed at around 340 °C, which is in agreement with the studies by [29,30].

The incorporation of CNTs into PLA resulted in a slight increase in the initial degradation temperature, suggesting that the CNTs act as a thermal stabilizer, providing a thermal protection effect [29,30,31]. This phenomenon is attributed to the barrier effect and the high thermal conductivity of the carbonaceous material.

The derivative thermogravimetric (DTG) analysis, illustrated in Figure 34, shows that the PLA/CNT composite undergoes decomposition at a higher temperature compared to neat PLA reported in the literature, indicating greater thermal stability in the initial stages of degradation. This result is in line with [32], which reported that carbon additives improve the thermal behavior of PLA due to an enhanced matrix–reinforcement interaction.

The overlapped ATD curves of specimens 01 and 07 exhibited similar decomposition stages, as both were based on a thermal decomposition process of mass loss that occurred in a single step. However, there was a slight difference between the peak heights. For specimen 11, a less pronounced peak was observed with a maximum rate value of T_peak max_ = 336.7 °C, while specimen 01 showed a more pronounced peak with T_peak max_ = 338.6 °C.

The specimens CP01 and CP11 have different CNT compositions (CP01 = 0.2%; CP11 = 0.6%) but exhibit the same injection temperatures (CP01 and CP11 = 190 °C). Both showed exothermic behavior.

For CP07, there is a less pronounced peak with a maximum rate value T_peak max_ = 316.74 °C, despite having similar CNT values (0.2%). The material’s extrusion temperature was higher compared to the others, T = 205 °C, which may have contributed to a lower peak. It was observed that the mass loss began between temperatures of 208 °C and 510 °C, which is lower than the other specimens.

### 3.8. Infrared Radiation by Fourier Transform (IRTF)

**Figure 35:**
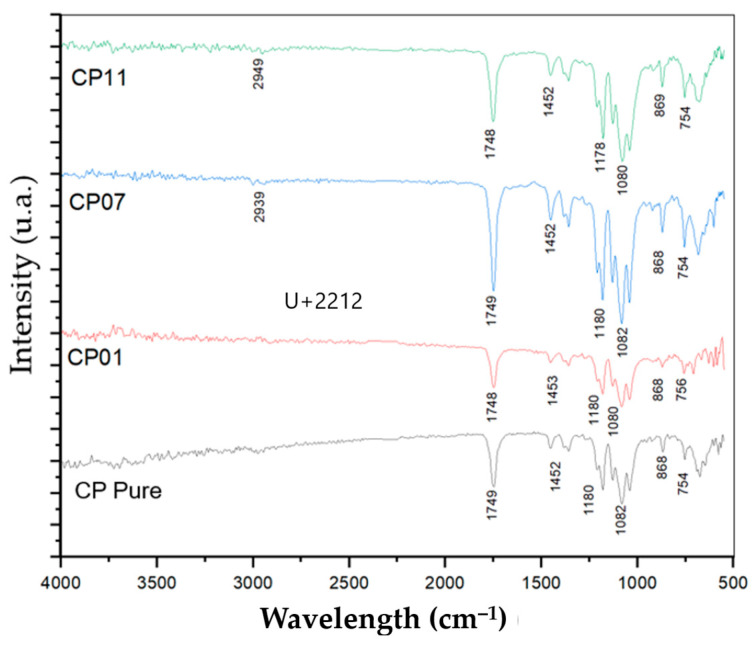
Infrared spectrum graph.

The Figure 35 displays the FTIR spectra obtained, where three characteristic absorption bands are observed: 2914–2993, 1744–1748, and 1181 cm^−1^ correspond to the stretching vibrations of the –OH, C–O, and CO groups of PLA, respectively. At 1453 cm^−1^, there is a bending vibration of CH3 groups, and at 1379–1362 cm^−1^ (not indicated), the bands arise from CH deformation and asymmetric/symmetric bending, respectively. The bands at 864 and 752 cm^−1^ correspond to the bending vibration of C–C and CH groups, respectively [22].

None of the FTIR spectra display the characteristic vibration of intermolecular –OH (3550–3200 cm^−1^) previously observed in CNT samples. A weak vibration at 2914 cm^−1^ associated with the presence of intramolecular –OH can be observed in the samples. Additionally, the stretching vibrations of the PLA CO groups at 1083 cm^−1^ are shifted to a lower wavenumber for PLA/CNT. This suggests that the adhesion mechanism between CNT and the PLA matrix may be associated with an interaction of terminal hydroxyl groups of PLA, included in carboxyl groups, with hydroxyl groups on the surface of CNT through a condensation reaction.

### 3.9. Finite Element Method (FEM) Simulation

The stress analyses on the propeller were conducted using the materials from Table 4, obtaining the propeller’s information during a flight simulation.

The propeller used for the simulation was of the 6045R type, corresponding to the QAV 250 drone, for which the mass was used to calculate the thrust. This drone has an average mass of 0.5 kg and four lifting propellers, which means each propeller supports 1/4 of the total mass, i.e., 125 g. This corresponds to each propeller bearing a load of approximately 1.23 N per entire propeller.

Using the calculated values above, the maximum stress on the propeller blades was obtained and compared with the stresses of the materials using simulation software. The value obtained for the von Mises stress was 15.4 MPa with a maximum displacement of 10.9 mm.

Considering values of 1.5 (commonly used in the development of radio-controlled aircraft) for the Safety Factor (S.F.) and 2.0 for the Load Factor (L.F.), as suggested in FAR-PART 25, it is necessary for the material to exhibit values exceeding 46.2 MPa. It is evident that the developed material can be used for the manufacturing of the propeller, taking into account the necessary precautions, as mentioned throughout the work, to ensure good print quality of the product, such as

- Good dispersion of nanoparticles in the PLA matrix;
- Adequate printing temperature;
- Printing orientation at 45°/−45°, as used in this research;
- Printing fill pattern.

**Figure 36:**
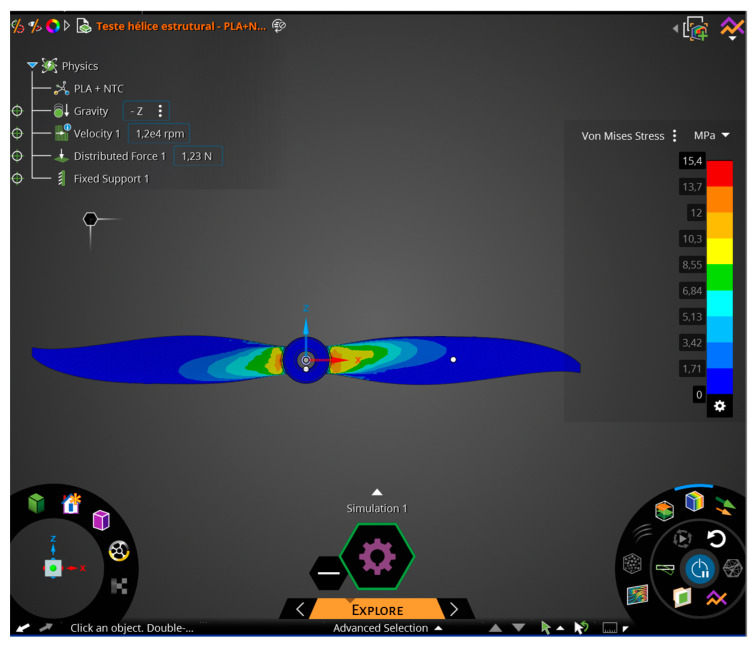
Finite element analysis (FEA) stress test results using Ansys Discovery.

**Figure 37:**
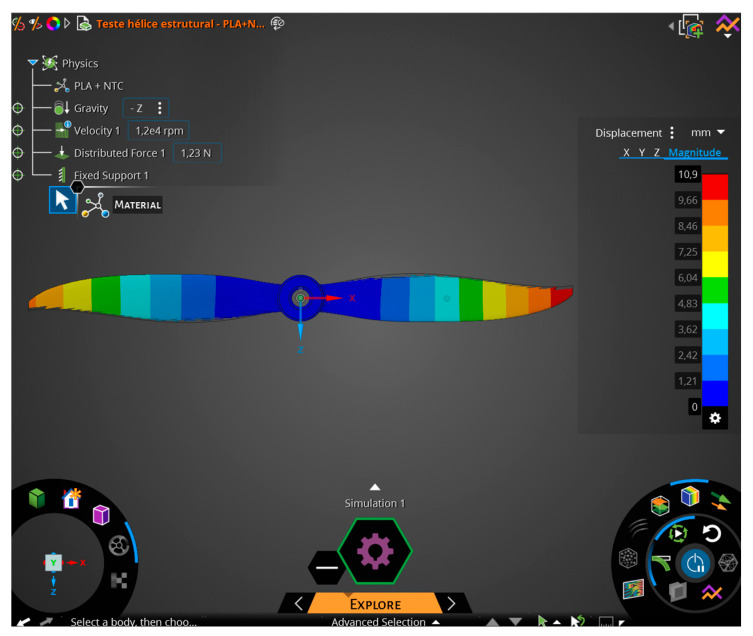
Finite element analysis (FEA) displacement test results using Ansys Discovery.

Figure 36 and Figure 37 depict the stress and displacement of the propeller blades when subjected to loads. The blue color represents the minimum values found, while the red color represents the maximum values found.

## 4. Conclusions

This study successfully demonstrated the potential of incorporating carbon nanotubes (CNTs) into a poly(lactic acid) (PLA) matrix, even when utilizing the additive manufacturing process with a 40% fill, for the production of high-performance 3D printing filaments. The research validated the effectiveness of Design of Experiments (DOE) to optimize the extrusion process, identifying the best conditions for achieving superior mechanical properties. Microscopy analysis and finite element simulation confirmed that the final material is a viable alternative for manufacturing drone propellers. In short, the work not only contributes to the advancement of composite materials but also offers a practical and innovative solution for the additive manufacturing sector, overcoming the limitations of pure PLA.

## Figures and Tables

**Table 1:** Experimental Design 23 (3 factors and 2 levels).

Factor 1: %CNT	Level 1: 0.2% Level 2: 1.0%
Factor 2: Temperature	Level 1: 190 °C Level 2: 220 °C
Factor 3: RPM	Level 1: 30 rpm Level 2: 60 rpm

**Table 2:** Full factorial worksheet.

C1	C2	C3	C4	C5	C6	C7
Standard Order	Run Order	Center Point	Blocks	%CNT	°C	RPM
3	1	1	1	0.2	220	30
1	2	1	1	0.2	190	30
11	3	0	1	0.6	205	45
10	4	0	1	0.6	205	45
4	5	1	1	1.0	220	30
9	6	0	1	0.6	205	45
7	7	1	1	0.2	220	60
6	8	1	1	1.0	190	60
2	9	1	1	1.0	190	30
5	10	1	1	0.2	190	60
8	11	1	1	1.0	220	60

**Table 3:** Response surface worksheet.

C1	C2	C3	C4	C5	C6	C7
Standard Order	Run Order	Center Point	Blocks	%CNT	°C	RPM
14	1	0	1	0.6	205	45
11	2	2	1	0.6	190	60
15	3	0	1	0.6	205	45
3	4	2	1	0.2	220	45
8	5	2	1	1.0	205	60
12	6	2	1	0.6	220	60
9	7	2	1	0.6	190	30
2	8	2	1	1.0	190	45
5	9	2	1	0.2	205	30
13	10	0	1	0.6	205	45
1	11	2	1	0.2	190	45
10	12	2	1	0.6	220	30
4	13	2	1	1.0	220	45
6	14	2	1	1.0	205	30
7	15	2	1	0.2	205	60

**Table 4:** Library with properties for the found nanocomposite.

Property	Value
Density	$1.9\;g/cm^{3}$
Young’s modulus	1.9 GPa
Poisson’s ratio	0.3
Shear modulus	0.729 GPa
Bulk modulus	1.58 GPa
Tensile yield strength	0
Tensile ultimate strength	46 GPa
Material	PLA + CNT

**Table 5:** Worksheet with the found response for tensile strength (MPa).

C1	C2	C3	C4	C5	C6	C7	C8
Standard Order	Run Order	Center Point	Blocks	%CNT	°C	RPM	Tension
3	1	1	1	0.2	220	30	17.07
1	2	1	1	0.2	190	40	16.14
11	3	0	1	0.6	205	45	26.45
10	4	0	1	0.6	205	45	28.85
4	5	1	1	1.0	220	30	18.16
9	6	0	1	0.6	205	45	27.30
7	7	1	1	0.2	220	60	27.13
6	8	1	1	1.0	190	60	23.29
2	9	1	1	1.0	190	30	24.49
5	10	1	1	0.2	190	60	27.34
8	11	1	1	1.0	220	60	22.57

**Table 6:** Response surface worksheet with tensile strength (MPa).

C1	C2	C3	C4	C5	C6	C7	C8	C9
Standard Order	Run Order	TypePt	Blocks	%CNT	°C	RPM	σ_T_ (MPa)	S (Standard Deviation)
14	1	0	1	0.6	205	45	20.96	4.1
11	2	2	1	0.6	190	60	33.29	5.21
15	3	0	1	0.6	205	45	23.32	4.04
3	4	2	1	0.2	220	45	30.47	1.48
8	5	2	1	1.0	205	60	31.23	3.61
12	6	2	1	0.6	220	60	31.16	1.76
9	7	2	1	0.6	190	30	32.01	2.9
2	8	2	1	1.0	190	45	32.63	3.42
5	9	2	1	0.2	205	30	22.87	1.68
13	10	0	1	0.6	205	45	24.92	4.8
1	11	2	1	0.2	190	45	48.87	4.59
10	12	2	1	0.6	220	30	30.05	4.04
4	13	2	1	1.0	220	45	32.43	3.52
6	14	2	1	1.0	205	30	20.04	4.79
7	15	2	1	0.2	205	60	34.89	1.42

**Table 7:** Thermal characteristics among the test specimens.

Material	Ti °C	T_(onset)_ °C	T_(endset)_ °C	Tf °C
**CP-01**	247.48	316.74	363.09	369.95
**CP-07**	226.65	286.50	341.67	346.49
**CP-11**	238.45	307.67	358.21	389.18

**Table 8:** Temperature versus percentage of degraded mass.

Material	T_(onset)_ °C	% Degraded Mass	T_(endset)_ °C	%Residue	T_f_ °C	%Residue
**CP-01**	316.7	81.3	363.1	7.05	670	0.80
**CP-07**	286.5	83.1	341.7	4.82	690	1.50
**CP-11**	307.7	84.3	358.2	7.24	638	1.54

**Table 9:** Curve Values for Test Specimens

Specimen	T_peak_ °C
**CP01**	338.6
**CP07**	316.7
**CP11**	336.7

## Data Availability

The original contributions presented in this study. Further inquiries can be directed to the corresponding author.
